# Recent Advances in Antimony Sulfide-Based Nanomaterials for High-Performance Sodium-Ion Batteries: A Mini Review

**DOI:** 10.3389/fchem.2022.870564

**Published:** 2022-04-07

**Authors:** Guangxin Wang, Mingyi Guo, Yunchao Zhao, Yibo Zhao, Kun Tang, Zhijun Chen, Heinz-Rolf Stock, Yong Liu

**Affiliations:** ^1^ Research Center for High Purity Materials, Henan University of Science and Technology, Luoyang, China; ^2^ Provincial and Ministerial Co-Construction of Collaborative Innovation Center for Non-Ferrous Metal New Materials and Advanced Processing Technology, Henan Key Laboratory of Non-Ferrous Materials Science and Processing Technology, School of Materials Science and Engineering, Henan University of Science and Technology, Luoyang, China; ^3^ Luoyang Bearing Research Institute Co., Ltd, Luoyang, China

**Keywords:** sodium-ion batteries, electrochemical performance, Sb_2_S_3_-based nanomaterials, anode materials, composites

## Abstract

Recently, sodium-ion batteries (SIBs) have attracted extensive attention as potential alternatives to lithium-ion batteries (LIBs) due to the abundance, even distribution, low cost, and environmentally friendly nature of sodium. However, sodium ions are larger than lithium ions so that the anode materials of LIBs are not suitable for SIBs. Therefore, many negative electrode materials have been investigated. Among them, Sb_2_S_3_-based nanomaterials have gradually become a research focus due to their high theoretical specific capacity, good thermal stability, simple preparation, and low price. In this review, the research progress of Sb_2_S_3_-based nanomaterials in the SIB field in recent years is summarized, including Sb_2_S_3_, Sb_2_S_3_/carbon composites, Sb_2_S_3_/graphene composites, and Sb_2_S_3_/M_x_S_y_ composites. Furthermore, the challenges and prospects for the development of Sb_2_S_3_-based nanomaterials are also put forward. We hope this review will contribute to the design and manufacture of high-performance SIBs and promote its practical application.

## Introduction

Recently, lithium-ion batteries (LIBs) have developed rapidly and are extensively used in electronic devices such as notebook computers, electric vehicles, and mobile phones (Qin et al., 2017; Chong et al., 2018; Schmuch et al., 2018; Pang et al., 2019; Yuan et al., 2019; Wang et al., 2020; Tao et al., 2022). Nevertheless, the distribution of lithium on earth is uneven, and its reserves are limited. In addition, there are still some problems that need to be solved for LIBs, such as poor low-temperature performance, safety problems, and high cost (Liu G. et al., 2018; Xing et al., 2020; Sui D. et al., 2021; Wang et al., 2021c; Shi et al., 2021). As a potential substitute for LIBs in energy storage devices, SIBs have attracted extensive attention because sodium is much cheaper than lithium, environmentally friendly, and SIBs show the same energy storage mechanism as LIBs (Wang et al., 2018; Cao et al., 2020; Sui et al., 2020). However, the ionic radius of sodium ion (Na^+^: 102 p.m.) is larger than that of lithium ion (Li^+^: 76 p.m.), which will lead to difficulties in the sodiation/desodiation process combined with a greater volume change. Consequently, electrode materials matched with LIBs are not suitable for SIBs (Zhao and Arumugam, 2015; Wang et al., 2017; Liu Q. et al., 2019; Liu Y. et al., 2019; Hao et al., 2019; Sui et al., 2020). Therefore, it is critical to investigate SIB electrode materials with high reversible capacity and excellent cycle stability.

As an important type of electrode material for SIBs, anode materials have been widely studied (Tao et al., 2021). Until now, considerable achievements have been made in the research of SIB anode materials, such as layered transition metal oxides (Xiong et al., 2011; Ma et al., 2020; Li Y. et al., 2020), polyanionic compounds (Li et al., 2015; Yu et al., 2018; Guo et al., 2020; Sui Y. et al., 2021), metal sulfide composites (Cui et al., 2018; Zhao et al., 2020), or alloy composites (Liu et al., 2016; Tao et al., 2021)*.* Metal sulfide anodes have a higher sodium storage capacity, and generally have lower redox potential, better electrochemical reversibility, and longer cycle life than metal oxides in charge/discharge reaction (Xie et al., 2018; Liu G. et al., 2019; Xu et al., 2019; Yao et al., 2019; Shan et al., 2020). Among them, Sb_2_S_3_ has a high theoretical capacity of 946 mA h g^−1^, and it is cheap and harmless to the environment (Zhu et al., 2015; Xie F. et al., 2019). Moreover, by combining the conversion reaction (Eq. 1) and alloying reaction (Eq. 2) between Na and S, Sb_2_S_3_ can produce a high-capacity anode and effectively play the role of S–Na and Sb–Na nanocomposites in SIBs (Yu et al., 2013; Liu et al., 2017). The following is the generally proposed electrochemical reaction mechanism between ${\text{Sb}}_{2}{\text{S}}_{3}$ and ${\text{Na}}^{+}$ (Liu et al., 2017; Xie F. et al., 2019):

$${\text{Conversion reaction: Sb}}_{2}{\text{S}}_{3}{\;+\;6\text{Na}}^{+}{\;+\;6\text{e}}^{-}\;\to {2\text{Sb }+\;3\text{Na}}_{2}\text{S}\text{.}$$ (1)

$${\text{Alloying reaction: }2\text{Sb }+\;6\text{Na}}^{+}{\;+\;6\text{e}}^{-}\;\to {2\text{Na}}_{3}\text{S}\text{.}$$ (2)

Sb_2_S_3_-based anode materials, such as multi-shell hollow Sb_2_S_3_ (Xie F. et al., 2019), Sb_2_S_3_/graphene composites (Li C.-Y. et al., 2017; Zhao et al., 2021), Sb_2_S_3_@FeS_2_/N-graphene (SFS/C) (Cao et al., 2020), and L-Sb_2_S_3_/Ti_3_C_2_ composites (He et al., 2021), have been reported in the application field of SIBs. For instance, Xiong et al. reported about Sb_2_S_3_ with nanostructure on S-doped graphene sheets for high-performance anode materials of SIBs (Xiong et al., 2016). Based on the interaction of heterogeneous interfaces between different components of metal sulfide, Cao et al. reported Sb_2_S_3_@FeS_2_ with heteroatom-doped graphene as a superior SIB anode material (Cao et al., 2020). Xu et al. (2019) reviewed updated research on multiple phase transformation mechanisms and strategies to improve the performance of Sb- and Bi-based chalcogenides for SIBs. Liu et al. reviewed recent studies on Sb-based electrode materials for applications, storage mechanisms, and synthesis strategies in SIBs, LIBs, and LMBs (liquid metal batteries) (Liu Z. et al., 2018). However, so far as we know, critical reviews that focus on Sb_2_S_3_-based electrode nanomaterials specifically for SIBs have rarely been reported.

**FIGURE 1:**
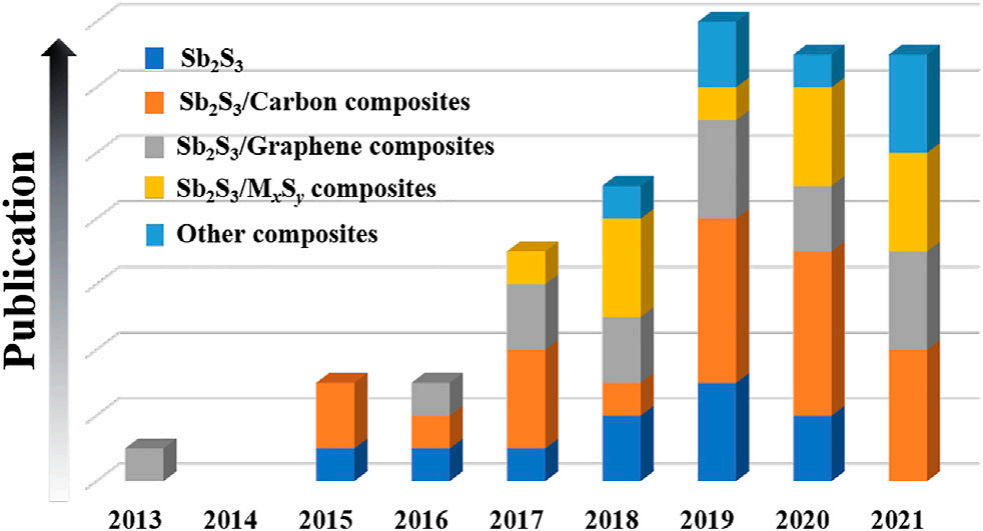
Bar chart of Sb_2_S_3_-based nanomaterials as anodes for SIBs in recent years.

Herein, the research achievements and progresses of Sb_2_S_3_-based nanomaterials for SIBs in recent years are summarized (see Figure 1). In addition, some rational suggestions on the research and design of Sb_2_S_3_-based nanomaterials for SIBs in the future are also presented. Finally, we hope that this review can attract more attention and promote the practical applications of Sb_2_S_3_-based nanomaterials in the SIB field.

## Research Progress of Sb_2_S_3_-Based Nanomaterials in High-Performance SIBs

Sb_2_S_3_ has advantages of low price, simple preparation, and good thermal stability (Xie F. et al., 2019; Cao et al., 2020). It is promising to be used as anode materials for high-capacity SIBs. A variety of Sb_2_S_3_-based anode materials have been reported. These are listed in Table 1.

**TABLE 1:** Electrochemical performances of Sb_2_S_3_-based nanomaterials as anodes for SIBs.

Materials	Initial	Capacity [mAh g^−1^/Cycles]	Rate capability [mAh g^−1^]	Ref
	Coulomb			
	Efficiency [%]			
Sb_2_S_3_				
Sb_2_S_3_	72.4	195 (200) at 0.1 A g^−1^	−	Fu et al. (2019)
Amorphous Sb_2_S_3_	65	512 (100) at 0.05 A g^−1^	534 at 3 A g^−1^	Hwang et al. (2016)
Sb_2_S_3_ micro tubes	37.1	201 (20) at 0.1 A g^−1^	286 at 0.2 A g^−1^	Jin Pan et al. (2017)
Colloidal Sb_2_S_3_	−	580 (100) at 0.3 A g^−1^	620 at 1.2 A g^−1^	Kravchyk et al. (2020)
Single crystal Sb_2_S_3_	50	579 (50) at 0.1 A g^−1^	358 at 1 A g^−1^	Pan et al. (2018a)
Sb_2_S_3_ hollow microspheres	62	384 (50) at 0.2 A g^−1^	386 at 2 A g^−1^, 314 at 3 A g^−1^	Xie et al. (2018)
Multi-shell Sb_2_S_3_	55	909 (50) at 0.1 A g^−1^	725 at 1 A g^−1^,604 at 2 A g^−1^	Xie et al. (2019a)
2D-Sb_2_S_3_	-	500 (100) at 0.2 A g^−1^	300 at 2 A g^−1^	Yao et al. (2019)
Sb_2_S_3_	77.6	38.6 (200) at 0.1 A g^−1^	109.5 at 1 A g^−1^, 95.1 at 2 A g^−1^	Zhao et al. (2020)
Flower-like Sb_2_S_3_	72.9	641.7 (100) at 0.2 A g^−1^	597.9 at 1A g^−1^, 554.6 at 2 A g^−1^	Zhu et al. (2015)
Sb_2_S_3_/carbon composites				
Sb_2_S_3_@YP-43%	42.6	736.2 (100) at 0.23 A g^−1^	476.5 (1,000) at 1.2 A g^−1^	Chang et al. (2020b)
Sb_2_S_3_/P/C	79	611 (100) at 0.05 A g^−1^	390 at 2 A g^−1^	Choi et al. (2016)
Sb_2_S_3_/C	78	538 (100) at 0.2 A g^−1^	579 at 0.5A g^−1^, 557 at 1 A g^−1^	Choi et al. (2017)
Sb_2_S_3_@C	38.2	267 (100) at 0.1 A g^−1^	283 at 1 A g^−1^	Dashairya et al. (2021)
Sb_2_S_3_/SCS	68.8	455.8 (100) at 0.1 A g^−1^	392 (15) at 0.5 A g^−1^, 263 (20) at 1 A g^−1^	Deng et al. (2019)
Sb_2_S_3_@N-C	80	765 (10) at 0.1 A g^−1^	625 (1,000) at 1 A g^−1^	Dong et al. (2019)
Sb_2_S_3_@C rods	68.5	699.1 (100) at 0.1 A g^−1^	578 at 1.5A g^−1^, 429 at 3.2 A g^−1^	Hongshuai Hou et al. (2015)
Sb_2_S_3_/C	−	545.6 (100) at 0.2 A g^−1^	550.8 (70) at 0.2 A g^−1^	Ge et al. (2018)
M-Sb_2_S_3_@DC	−	326 (100) at 0.5 A g^−1^	451 at 1 A g^−1^,366 at 3 A g^−1^	Ge et al. (2020)
Sb_2_S_3_/CM	64.7	426 (150) at 0.1 A g^−1^	−	Jaramillo-Quintero et al. (2021)
Sb_2_S_3_/Sb-CM	67.1	608 (150) at 0.1 A g^−1^	−	Jaramillo-Quintero et al. (2021)
Sb_2_S_3_/S-CM	66.9	675 (150) at 0.1 A g^−1^	552 at 1 A g^−1^, 481 at 2 A g^−1^	Jaramillo-Quintero et al. (2021)
Sb_2_S_3_@CNTs	66.4	732 (110) at 0.05 A g^−1^	668 at 1 A g^−1^, 584 at 2 A g^−1^	Jiang et al. (2021)
Sb_2_S_3_@MWCNTs	79.2	412.3 (50) at 0.05 A g^−1^	368.8 at 0.5 A g^−1^, 339.1 at 1 A g^−1^	Li et al. (2017b)
Amorphous Sb_2_S_3_/CNT	77.8	704 (50) at 0.1 A g^−1^	601 at 2 A g^−1^,474 at 3 A g^−1^	Li et al. (2019)
Sb_2_S_3_/CFC	76	736 (650) at 0.5 A g^−1^	649 (400) at 2 A g^−1^, 585 (400) at 5 A g^−1^	Liu et al. (2017)
CPC/Sb_2_S_3_	80	443 at 0.1 A g^−1^	220 (200) at 1 A g^−1^	Mullaivananathan and Kalaiselvi, (2019)
Sb_2_S_3_/CS	60	321 (200) at 0.2 A g^−1^	221 at 5 A g^−1^	Xie et al. (2019b)
Sb_2_S_3_@CNF	57.4	267.8 (100) at 0.1 A g^−1^	221 at 1 A g^−1^,178 at 5 A g^−1^	Zhai et al. (2020)
Sb_2_S_3_@NCFs	56.5	412 (50) at 0.05 A g^−1^	291 at 1 A g^−1^, 244 at 2 A g^−1^	Zhang et al. (2021b)
SS/Sb@C-1	70.9	171 (200) at 0.1 A g^−1^	253.2 at 1A g^−1^, 202.8 at 2 A g^−1^	Zhao et al. (2020)
SS/Sb@C-2	66.4	474.6 (200) at 0.1 A g^−1^	367 (150) at 1 A g^−1^,311.1 (150) at 2 A g^−1^	Zhao et al. (2020)
Sb_2_S_3_/graphite	84	733 at 0.1 A g^−1^	656 (100) at 1 A g^−1^, 495 (100) at 10 A g^−1^	Zhao. and Manthiram, (2015)
Sb_2_S_3_/graphene composites				
SN-RGO/Sb_2_S_3_	57	507 (150) at 0.1 A g^−1^	443.46 at 1 A g^−1^, 364.89 at 2 A g^−1^	Bag et al. (2019)
Sb_2_S_3_/RGO	55.9	262 (100) at 0.1 A g^−1^	210 at 1 A g^−1^	Dashairya et al. (2021)
Sb_2_S_3_/RGO	75.6	220 (50) at 0.05 A g^−1^	−	Dashairya and Saha, (2020)
Sn@Sb_2_S_3_-RGO	69.8	597.6 (60) at 0.2 A g^−1^	541 (70) at 0.5 A g^−1^	Deng et al. (2018)
Sb_2_S_3_/RGO	66.4	555 (70) at 0.1 A g^−1^	−	Fan and Xie, (2019)
Sb_2_S_3_/graphene	−	760 (100) at 0.05 A g^−1^	420 (100) at 1.5 A g^−1^	Li et al. (2017a)
Sb_2_S_3_/RGO	−	687.7 (80) at 0.05 A g^−1^	495.1 (80) at 0.2 A g^−1^,414.8 (100) at 0.5 A g^−1^	Pan et al. (2018b)
Sb_2_S_3_/RGO	52.6	652 (60) at 0.1 A g^−1^	527 at 1 A g^−1^, 381 at 2 A g^−1^	Wen et al. (2019)
Sb_2_S_3_/RGO	85.7	581.2 (50) at 0.05 A g^−1^	309.8 (10) at 2 A g^−1^	Wu et al. (2017)
Sb_2_S_3_/SGS	−	524.4 (900) at 2 A g^−1^	591.6 at 5 A g^−1^	Xiong et al. (2016)
RGO/Sb_2_S_3_	69.2	670 (50) at 0.05 A g^−1^	611 (5) at 1.5 A g^−1^, 520 (5) at 3 A g^−1^	Yu et al. (2013)
Sb_2_S_3_@N-C/RGO	57.6	368 (200) at 0.2 A g^−1^	338 at 1 A g^−1^, 253 at 5 A g^−1^	Zhan et al. (2021)
Sb_2_S_3_–graphene	55.9	881.2 (50) at 0.1 A g^−1^	536.4 at 1 A g^−1^	Zhao et al. (2021)
S-RGO/Sb_2_S_3_	63.9	509 (200) at 0.1 A g^−1^	239 (2000) at 5 A g^−1^	Zhou et al. (2020b)
Sb2S3/MxSy composites				
Sb_2_S_3_@FeS_2_/N-graphene (SFS/C)	82.4	725.4 at 0.1 A g^−1^	645.6 at 1A g^−1^, 564.3 at 5 A g^−1^	Cao et al. (2020)
Sb_2_S_3_-SnS_2_	77.9	616 (50) at 0.5 A g^−1^	510 at 10 A g^−1^	Fang et al. (2019)
In_2_S_3_-Sb_2_S_3_@MCNTs	−	454 (40) at 0.2 A g^−1^	402 at 1.6 A g^−1^,355 at 3.2 A g^−1^	Huang et al. (2018)
Sb_2_S_3_/MoS_2_ NWs	82.9	800 at 0.1 A g^−1^	570 at 3.2 A g^−1^	Li P. et al. (2020)
Sb_2_S_3_-Bi_2_S_3_@C@RGO	68.1	600.7 (150) at 1 A g^−1^	514.5 at 5 A g^−1^, 485.8 at 8 A g^−1^	Li et al. (2021)
Sb_2_S_3_@SnS@C	79	516 (100) at 0.1 A g^−1^	442 (200) at 1 A g^−1^, 200 (1,300) at 5 A g^−1^	Lin et al. (2021)
ZnS-Sb_2_S_3_@C	61.4	630 (120) at 0.1 A g^−1^	390.6 at 0.8 A g^−1^	Dong et al. (2017)
SnS_2_/Sb_2_S_3_@RGO	82.3	642 (100) at 0.2 A g^−1^	593 at 2 A g^−1^, 567 at 4 A g^−1^	Wang et al. (2018)
Sb_2_S_3_/MoS_2_@C (SMS@C)	79.5	623.2 at 0.1 A g^−1^	465.6 (100) at 1 A g^−1^, 411.5 (650) at 5 A g^−1^	Wang et al. (2021a)
Sb_2_S_3_/MoS_2_	75.9	568.4 at 0.1 A g^−1^	423.2 (100) at 1 A g^−1^	Wang et al. (2021a)
Sb_2_S_3_/MoS_2_	48.5	561 (100) at 0.1 A g^−1^	628 at 1A g^−1^, 507 at 2 A g^−1^	Zhang et al. (2018)
α-Sb_2_S_3_@CuSbS_2_	82.2	506.7 (50) at 0.05 A g^−1^	293 at 3 A g^−1^	Zhou et al. (2020a)
Other composites				
Sb_2_S_3_@SnO_2_	54.2	582.9 (100) at 0.05 A g^−1^	441.6 at 1A g^−1^, 237.1 at 5 A g^−1^	Chang et al. (2020a)
L-Sb_2_S_3_/Ti_3_C_2_	65.7	445.5 (100) at 0.1 A g^−1^	339.5 at 2 A g^−1^	He et al. (2021)
Sb_2_S_3_@Ti_3_C_2_T_x_		329 (100) at 0.1 A g^−1^	118 (500) at 2 A g^−1^	Ren et al. (2021)
Sb_2_S_3_@PPy	63.7	881 (50) at 0.1 A g^−1^	390 (400) at 2 A g^−1^	Shi et al. (2019)
Sb_2_S_3_/MMCN@PPy	−	446 (50) at 0.1 A g^−1^	269 (300) at 1 A g^−1^	Yin et al. (2019)
Sb_2_S_3_@m-Ti_3_C_2_T_x_	51	156 (100) at 0.1 A g^−1^	72 (1000) at 2 A g^−1^	Zhang et al. (2021a)
Sb_2_S_3_/PPy	70	427 (50) at 0.1 A g^−1^	236 (50) at 0.5 A g^−1^	Zheng et al. (2018)

Notes: 2D-Sb_2_S_3_ = two-dimensional Sb_2_S_3_; Sb_2_S_3_@YP-43% = 43% contents Sb_2_S_3_ mixed with YP80F active carbon (YP); Sb_2_S_3_/SCS, stibnite/sulfur-doped carbon sheet; M-Sb_2_S_3_@DC, metal-sulfides with double carbon; CM, carbon matrix; CNTs, carbon nanotubes; MWCNTs, multiwalled carbon nanotubes; CFC, carbon fiber cloth; CPC, coir pith derived carbon; Sb_2_S_3_/CS, Sb_2_S_3_ embedded in carbon–silicon oxide nanofibers; CNF, multichannel N-doped carbon nanofiber; NCFs = N-doped 3D carbon nanofibers; RGO, reduced graphene oxide; Sb_2_S_3_/SGS, Sb_2_S_3_/sulfur-doped graphene sheets; SN-RGO/Sb_2_S_3_ = sulfur, nitrogen dual doped RGO/Sb_2_S_3_; Sb_2_S_3_@N-C/RGO, Sb_2_S_3_/nitrogen-doped carbon/RGO; S-RGO/Sb_2_S_3_ = sulfur-doped RGO/Sb_2_S_3_; MCNTs, multiwalled carbon nanotubes; Sb_2_S_3_/MoS_2_ NWs, Sb_2_S_3_/MoS_2_ core-shell nanowires; PPy, polypyrrole.

### Sb_2_S_3_

To obtain Sb_2_S_3_ anodes with high energy density and capacity in SIBs, researchers prepared Sb_2_S_3_ with different morphologies, such as amorphous Sb_2_S_3_ (Hwang et al., 2016), flower-like Sb_2_S_3_ (Zhu et al., 2015), multi-shell Sb_2_S_3_ (Xie F. et al., 2019), or Sb_2_S_3_ hollow microspheres (Xie et al., 2018).

For example, Hwang et al. (2016) synthesized aspherical, amorphous α-Sb_2_S_3_ *via* a facile polyol route at room temperature, which is different from the previous routes of forming crystalline Sb_2_S_3_ at high temperature (mainly, hydrothermal method) (Zhu et al., 2015). As shown in Supplementary Figure S1A, α-Sb_2_S_3_ nanoparticles were composed of spherical aggregates of sub-component nanoparticles with diameters of 150–300 nm. When investigated as SIB anodes, the α-Sb_2_S_3_ nanoparticle electrode displayed a charge capacity of 512 mA h g^−1^ after 100 cycles at a current density of 50 mA g^−1^, and showed a better cycle performance and excellent rate performance, in contrast with the commercial crystal Sb_2_S_3_ electrode (Supplementary Figure S1B).

Moreover, two-dimensional (2D) nanomaterials with large surface area and ultrafine thickness have attracted more and more attention. For instance, Yao et al. (2019) designed 2D-Sb_2_S_3_ nanosheets by using a facile and scalable Li intercalation assisted stripping method. The 2D-Sb_2_S_3_ nanosheets (2D-SS) showed a good layered structure with a mean thickness of 3.8 nm (Supplementary Figure S1C). The large pore volume and large surface area of 2D-SS nanosheets are beneficial to the electrolyte penetration and the volume change during cycles. Therefore, 2D-SS nanosheet anodes showed remarkable rate capability and stable cycle performance in both SIBs and LIBs. When used in SIBs (Supplementary Figure S1D), the 2D-SS anode displayed a superior capacity of ∼500 mA h g^−1^ after 100 cycles at 200 mA g^−1^ current rate.

Recently, Sb_2_S_3_ materials with three-dimensional (3D) hierarchical architecture were designed and synthesized to expand the contact surface area of the electrode and electrolyte and adapt it to volume expansion (Jin Pan et al., 2017; Xie et al., 2018; Xie F. et al., 2019). Xie et al. (2018) used SbCl_3_ and _L_-cysteine as raw materials and successfully synthesized Sb_2_S_3_ hollow microspheres by a hydrothermal method. The SEM image and cycling performance of Sb_2_S_3_ hollow microspheres are shown in Supplementary Figures S1E,F. However, large internal voids in hollow structures can greatly reduce bulk energy density. In order to obtain a high volumetric energy density and maintain a high gravimetric energy density, Xie F. et al. (2019) synthesized multi-shell hollow Sb_2_S_3_ structures using the metal-organic framework templates (MOFs) (Supplementary Figure S1G). Used as an anode in SIBs (Supplementary Figure S1H), the multi-shell Sb_2_S_3_ exhibited reversible capacities of 909, 806, 725, and 604 mA h g^−1^ at various currents of 100, 400, 1,000, and 2,000 mA g^−1^, respectively, higher than the single-shell Sb_2_S_3_ structure.

### Sb_2_S_3_/Carbon Composites

Carbon materials have received considerable attention because of their superior characteristics, such as large specific surface area, high conductivity, excellent flexibility, and chemical stability (Tao et al., 2021). During the use of SIBs, Sb_2_S_3_ will undergo transformation and alloying reaction, which results in excessive volume expansion/contraction of the material, and hinders the application of Sb_2_S_3_ energy storage effect. Therefore, Sb_2_S_3_ is usually combined with carbon materials to inhibit the volume change, such as Sb_2_S_3_/carbon-rods (Hongshuai Hou et al., 2015), Sb_2_S_3_/carbon-nanotubes (Li J. et al., 2017; Li et al., 2019), Sb_2_S_3_/carbon-nanofiber (Zhai et al., 2020; Zhang Q. et al., 2021), or Sb_2_S_3_/heteroatom-doped carbon (Dong et al., 2019; Jaramillo-Quintero et al., 2021).

For instance, Hongshuai Hou et al. (2015) designed one-dimensional (1D) Sb_2_S_3_@C rods as a distinctive anode material to improve the electrochemical performance of SIBs *via* a solvothermal method (Supplementary Figure S2A). The Sb_2_S_3_@C rod electrode could deliver 699.1 mA h g^−1^ at a current rate of 100 mA g^−1^ after 100 cycles (Supplementary Figure S2B). Liu et al. (2017) reported a hydrothermal method for preparing Sb_2_S_3_ micro-nanospheres loaded on carbon fiber cloth (CFC). The obtained composite materials were denoted as SS/CFC. The flexible carbon fiber cloth was completely covered by spherical Sb_2_S_3_ in Supplementary Figure S2C, which could greatly accommodate the volume change (Guo et al., 2019). When used as electrodes for SIBs (Supplementary Figure S2D), SS/CFC electrodes exhibited an excellent initial discharge capacity of 1,048 mA h g^−1^ at 0.5 A g^−1^, and displayed a reversible capacity of 736 mA h g^−1^ after 650 cycles in the voltage range of 0.01–2.00 V. After two initial cycles, the corresponding Coulombic efficiency of SS/CFC rapidly increased to ∼100%.

To boost the storage performance of SIBs, Sb_2_S_3_ can be combined with carbon doped with heteroatoms (e.g., N, S, P, and Sb), thus improving the conductivity, the storage regions, and the active sites (Choi et al., 2016; Dong et al., 2019; Zhai et al., 2020; Jaramillo-Quintero et al., 2021). For instance, Zhao et al. (2020) utilized the oxygen-function group of phenolic resin and constructed Sb_2_S_3_ with hierarchical interfaces (antimony and sulfur-doped carbon) (Supplementary Figure S2E). The final obtained composites were denoted as SS/Sb@C. When evaluated as electrode materials for SIBs (Supplementary Figure S2F), SS/Sb@C delivered a reversible capacity of 474.6 mA h g^−1^ and a capacity retention rate of 97.1% after 200 cycles at 0.1 A g^−1^, showing better cyclic stability and superior rate capability than those of the Sb_2_S_3_ anodes without heteroatoms (38.6 mA h g^−1^). This was due to the double control synergy of Sb-shell structure and S-doped carbon structure, which effectively expanded the polysulfide diffusion path, enhanced the reversibility of conversion reaction, and thus improved the Na-storage capacity of SIBs (Yu et al., 2020; Wang et al., 2021b). This kind of reasonable design was expected to bring bright prospects for the design of metal sulfides as advanced anodes of SIBs.

### Sb_2_S_3_/Graphene Composites

Graphene has high specific surface area, which is convenient for constructing interconnected pore structures to form conductive networks. In addition, it can also provide a platform for the growth of active materials (Lv et al., 2016; Sui et al., 2020; Wang X. et al., 2021; Liu et al., 2021). The combination of Sb_2_S_3_ with graphene can provide excellent Na^+^ energy storage properties. Therefore, many composites have been designed in recent years, such as Sb_2_S_3_/RGO (RGO = reduced graphene oxide) (Yu et al., 2013; Wen et al., 2019), Sn@Sb_2_S_3_-RGO (tin assisted Sb_2_S_3_ decorated on RGO) (Deng et al., 2018), S-RGO/Sb_2_S_3_ (sulfur-doped RGO-based composite with Sb_2_S_3_) (Zhou X. et al., 2020), and Sb_2_S_3_/N-C/RGO (Sb_2_S_3_@nitrogen-doped carbon decorated on RGO) (Zhan et al., 2021), to improve the storage properties of SIBs.

**FIGURE 2:**
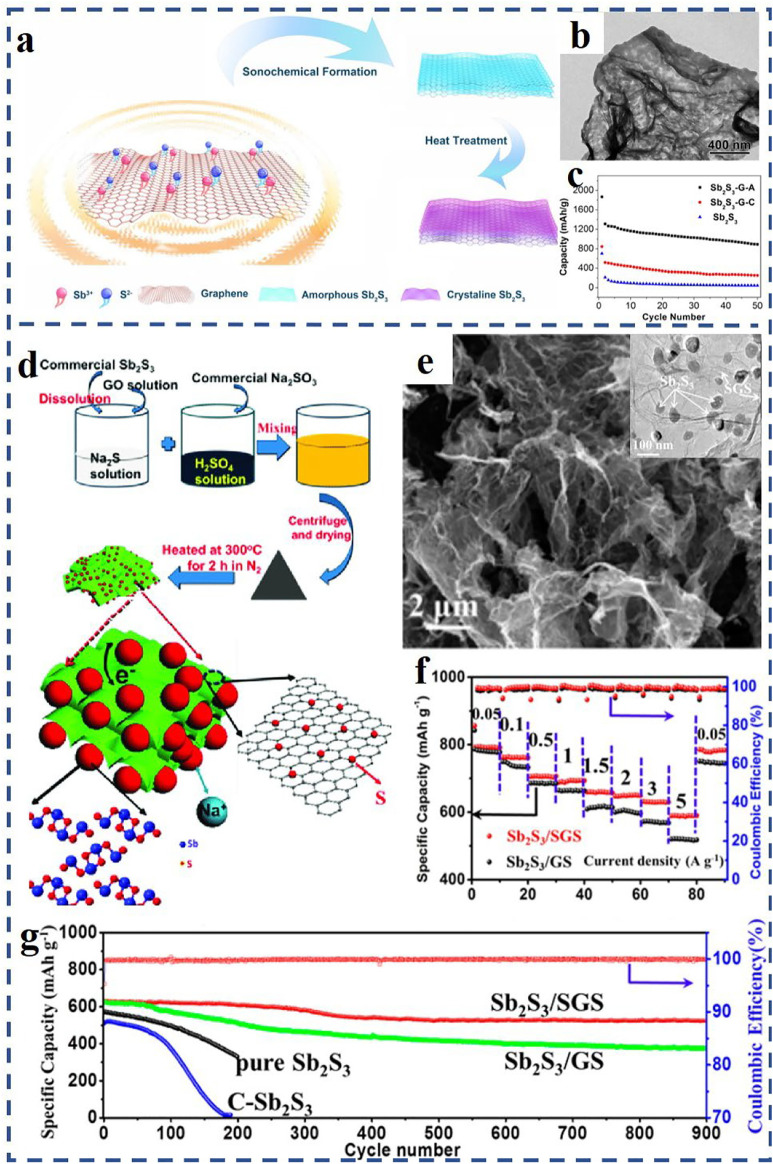
**(A)** Schematic illustration of the preparation process of the amorphous and crystalline Sb_2_S_3_/graphene composites; **(B)** TEM image of the amorphous Sb_2_S_3_–graphene composites; **(C)** cycle performances of the pristine Sb_2_S_3_ and amorphous and crystalline Sb_2_S_3_–graphene electrodes (denoted as Sb_2_S_3_-G-A and Sb_2_S_3_-G-C); **(D)** formation process of the Sb_2_S_3_/S-doped graphene nanocomposite (Sb_2_S_3_/SGS); **(E)** SEM and TEM images of the Sb_2_S_3_/SGS nanocomposite; **(F)** rate performances of the Sb_2_S_3_/SGS electrode and Sb_2_S_3_–graphene electrode (Sb_2_S_3_/GS) under different current density; **(G)** cycle performances of three experimental electrodes at 2 A g^−1^. **(A–C)** Reproduced with permission from Zhao et al. (2021). Copyright 2020, Elsevier. **(D–G)** Reproduced with permission from Xiong et al. (2016), Copyright 2016, American Chemical Society.

For example, Yu et al. (2013) received a uniform coating of Sb_2_S_3_ on RGO (RGO/Sb_2_S_3_) through a solution-based synthesis method and applied it as SIB anode materials. The RGO/Sb_2_S_3_ composite with a small particle size of 15–30 nm allows Na^+^ to move in and out of the particles rapidly during charge and discharge process. In addition, the 2D-layered structure of graphene and Sb_2_S_3_ can form oriented layered composites with excellent properties. Compared with traditional synthesis techniques, the ultrasound sonochemical method can create particular reaction conditions, and make it possible to prepare nanostructured materials with special properties by acoustic cavitation effects. Zhao et al. (2021) synthesized a special amorphous nanostructure composite material of Sb_2_S_3_/graphene by an ultrasound sonochemical synthesis technique (Figure 2A). As can be seen from Figure 2B, Sb_2_S_3_ nanoparticles were tightly covered on the graphene nanosheets and evenly distributed on both sides. The Sb_2_S_3_/graphene nanocomposites with amorphous structure had good tolerance and adaptability to drastic volume changes. Compared to the crystalline counterpart (Li C.-Y. et al., 2017), the amorphous Sb_2_S_3_/graphene nanocomposite displayed a superior electrochemical property with a higher reversible capacity of 881.2 mA h g^−1^ at 0.1 A g^−1^ after 50 cycles (Figure 2C).

Furthermore, doping heteroatoms (e.g., N, P, S, Sn) on graphene-based materials by surface chemical modification can effectively improve the properties of SIBs (Xiong et al., 2016; Deng et al., 2018; Zhou X. et al., 2020; Zhan et al., 2021). For example, Xiong et al. (2016) obtained a unique Sb_2_S_3_/S-doped graphene anode material (denoted as Sb_2_S_3_/SGS) *via* firm chemical binding of nano-Sb_2_S_3_ structure on S-doped graphene nanosheets (SGS). Schematic illustration of the preparation process of the Sb_2_S_3_/SGS composite is displayed in Figure 2D. As shown in Figure 2E, Sb_2_S_3_ nanoparticles are wrapped by flexible SGS and exhibit a size of 30–80 nm. When tested at 0.05 A g^−1^ current rate, the Sb_2_S_3_/SGS anode reaches a high specific capacity of 792.8 mA h g^−1^ after 90 cycles (see Figure 2F). After 900 cycles at a higher current rate of 2 A g^−1^ (in Figure 2G), the Sb_2_S_3_/SGS anode still has an excellent cycle life, and the capacity retention rate is ∼83%.

### Sb_2_S_3_/M_*x*_S_*y*_ Composites

Most metal sulfides (M_*x*_S_*y*_) have hierarchical structures, and Na^+^ can easily move in the interlayers of metal sulfides without damaging their hierarchical structures (Tao et al., 2021). Thus, the use of binary metal sulfides to construct heterostructures to reduce the huge internal stress of alloy-based anodes and maintain the integrity of nanostructures has attracted extensive attention (Wang et al., 2018; Lin et al., 2021; Wang et al., 2019a). In this context, common metal sulfides (M_*x*_S_*y*_), including SnS_2_ (Wang et al., 2018), ZnS (Dong et al., 2017), FeS_2_ (Cao et al., 2020), In_2_S_3_ (Huang et al., 2018), and Bi_2_S_3_ (Li et al., 2021), have been combined with Sb_2_S_3_ as anode materials of SIBs.

For example, a composite of multiwalled carbon nanotubes (MCNTs) and In_2_S_3_-Sb_2_S_3_ particles (denoted as I-S@MCNTs) with a unique morphology of formicary microspheres was formed to solve the poor cycling stability and rate performance of SIBs (Huang et al., 2018). As shown in Supplementary Figure S3A, the hierarchical spheres are assembled by crumpled nanosheets (5–8 nm), which significantly shorten the diffusion path and accelerate the transport rate of Na^+^. Similarly, Wang D. et al. (2021) designed an armored hydrangea-like Sb_2_S_3_/MoS_2_ heterostructure composite (denoted as SMS@C) as a superior SIB anode material (Supplementary Figure S3B). After 650 cycles at a higher current density of 5 A g^−1^, the SMS@C anode exhibited an enhanced cycling performance of 411.5 mA h g^−1^ (Supplementary Figure S3E). Additionally, Dong et al. (2017) designed a polyhedron composite (∼1.5 μm) with a ZnS inner-core structure and Sb_2_S_3_/C double-shell structure (ZnS-Sb_2_S_3_@C), capitalizing on full advantages of the zeolitic imidazolate framework (ZIF-8). The structure of ZnS-Sb_2_S_3_@C core-double shell composites had enough space to greatly adapt to the volume expansion during the repeated insertion/extraction of Na^+^, and exhibited a superior reversible capacity of 630 mA h g^−1^ at a current density of 0.1 A g^−1^ after 120 cycles with a high Coulombic efficiency of ∼100% (Supplementary Figures S3C,F).

Recently, a breakthrough about Sb_2_S_3_@FeS_2_ hollow nanorods used as high-performance SIB electrode materials was reported. Cao et al. (2020) embedded Sb_2_S_3_@FeS_2_ hollow nanorods (SFS) into a nitrogen-doped graphene matrix, and synthesized Sb_2_S_3_@FeS_2_/N-doped graphene composite (denoted as SFS/C) *via* a simple two-step solvothermal synthesis technique (Supplementary Figures S3D,G). The clever design of the heterostructure extremely accelerated the Na^+^ transport, and greatly alleviated the volume expansion under long-period performance (1,000 cycles) (Wu et al., 2019a; Wu et al., 2019b; Liu et al., 2022). The SFS/C anode displayed a superior reversible capacity of 725.4 mA h g^−1^ after 90 cycles at 0.1 A g^−1^ (see Supplementary Figure S3H). When tested even at 5 A g^−1^, the SFS/C anode had an excellent cycle performance with a capacity retention of ∼85.7% after 1,000 cycles (Supplementary Figure S3I).

### Other Composites

In addition to the aforementioned Sb_2_S_3_-based nanomaterials, polypyrrole (PPy) (Wang et al., 2016; Zheng et al., 2018), MXene (M_n+1_X_n_T_x_, where M is the early transition metal, X represents C/N, and T_x_ is the surface functional group (-O, -OH or -F), n = 0,1,2,3,4. e.g*.*, Ti_3_C_2_T_x_, Ti_3_C_2_) (Wang et al., 2019b; Zhang H. et al., 2021; He et al., 2021), and metal oxides (e.g*.*, SnO_2_) (Chang et al., 2020a) can also be combined with Sb_2_S_3_ to fabricate better SIB anodes.

For instance, Shi et al. (Yin et al., 2019) prepared Sb_2_S_3_/meso@microporous carbon nanofibers@polypyrrole composites (denoted as Sb_2_S_3_/MMCN@PPy) though a novel multi-step method combining polymerization, sulfidation and solvothermal process (Supplementary Figure S4A). SEM image of Sb_2_S_3_/MMCN@PPy composites is shown in Supplementary Figure S4B. When investigated as SIB anode, Sb_2_S_3_/MMCN@PPy composite exhibited a discharge capacity of 535.3 mA h g^−1^ at a current density of 100 mA g^−1^, and the discharge specific capacity could recover to 446 mA h g^−1^ after 50 cycles when returned to 100 mA g^−1^ current rate (Supplementary Figure S4C). Shi et al. (2019) synthesized Sb_2_S_3_@PPy coaxial nanorods *via* a hydrothermal method. When tested at 100 mA g^−1^, it showed a superior reversible capacity as high as 881 mA h g^−1^ after 50 cycles, which was higher than those reported of MWNTs@Sb_2_S_3_@PPy composites (Wang et al., 2016), flower-like Sb_2_S_3_/PPy microspheres (Zheng et al., 2018), and Sb_2_S_3_/MMCN@PPy composites (Yin et al., 2019).

Furthermore, MXene is considered as an outstanding matrix because of the effective diffusion and mobility for Na^+^ and excellent electronic conductivity. Ti_3_C_2_T_x_ is one of the most studied MXene materials, and the theoretical capacity is 352 mA h g^−1^ when used as the anode of SIBs (Zhang H. et al., 2021; He et al., 2021; Ren et al., 2021). For instance, Zhang H. et al. (2021); Ren et al. (2021) prepared Sb_2_S_3_@Ti_3_C_2_T_x_ composite and Sb_2_S_3_@m-Ti_3_C_2_T_x_ composite by a wet chemical method, in which Sb_2_S_3_ nanoparticles were *in situ* nucleated and grown uniformly on the surface of Ti_3_C_2_T_x_ nanosheets. It was found that Ti_3_C_2_T_x_, as a conductive skeleton, could effectively alleviate the volume expansion of Sb_2_S_3_ during charge/discharge progress. In 2021, inspired by the stomatal structure from natural leaves, He et al. (2021) successfully synthesized Sb_2_S_3_/nitrogen-doped Ti_3_C_2_ composites (denoted as L-Sb_2_S_3_/Ti_3_C_2_) *via* a solvothermal method (Supplementary Figure S4D). L-Sb_2_S_3_/Ti_3_C_2_ composite showed a unique elm leaf-like morphology in Supplementary Figure S4E, with a length of 60–80 nm and a width of 30–40 nm, respectively. When used as SIB anode, L-Sb_2_S_3_/Ti_3_C_2_ composite displayed a high capacity of 502.2 mA h g^−1^ at a current rate of 100 mA g^−1^ from 0.01 to 3 V (Supplementary Figure S4F).

## Conclusion and Outlook

In this review, we briefly summarize the applications of Sb_2_S_3_-based nanomaterials for high-performance SIBs, mainly including Sb_2_S_3_, Sb_2_S_3_/carbon composites, Sb_2_S_3_/graphene composites, Sb_2_S_3_/M_*x*_S_*y*_ composites, and other related composites. Although many significant works have been made in SIBs, there are still some problems that need to be solved, and we propose some possible directions for the anode research of SIBs in the future:

1) During the charge/discharge cycles, Sb_2_S_3_ nanoparticles are easy to accumulate because of their high surface activity energy. This results in a significant volume change and capacity declining. Therefore, it is necessary to design and fabricate more reasonable nanostructures, such as hierarchical hollow nanotubes or hierarchical spheres (Xie F. et al., 2019), to fully buffer the strain of volume change and further improve the cycling performance. In addition, some soft materials could be added to improve the flexibility, so as to avoid the collapse of the anode due to the volume expansion.

2) Carbonaceous materials are often the main choice to combine with Sb_2_S_3_ to build dense conductive physical barriers. However, the content of Sb_2_S_3_ and the corresponding specific capacity of composite materials are reduced. Therefore, the carbon content should be optimized so that the Sb_2_S_3_-based materials achieve better electrochemical performance. In addition, Sb_2_S_3_/carbonaceous composites fabricated by traditional synthesis techniques suffer from the poor mechanical adhesion and high interface resistance between Sb_2_S_3_ and carbonaceous materials. It is highly desirable to optimize the preparation methods and explore more carbonaceous materials (e.g., biochar, amorphous carbon) to establish compact conductive physical barriers to further enhance the electrochemical performance of Sb_2_S_3_-based materials.

3) Until now, the cycle lives of many Sb_2_S_3_-based materials have been tested at room temperature. In order to satisfy the demands of different applications, it is very urgent to explore Sb_2_S_3_-based anode materials that can cycle under either higher temperature (up to 60 °C) or lower (−20°C).

4) The mechanism of Na^+^ storage in Sb_2_S_3_-based nanomaterials and the phase changes during repeated charging/discharging still need to be explored. Operating technologies, such as *in situ* X-ray technology, *in situ* scanning probe microscopy, technologies based on synchronized X-rays, as well as *in situ* electron microscopy, are very helpful in acquiring time-related information and studying the mechanism of Na^+^ storage of Sb_2_S_3_-based nanomaterials. Therefore, more research using operating technology is needed to deeply understand Sb_2_S_3_-based electrode nanomaterials used in SIBs.

## Supplementary Material

Supplementary Figure S1
**(A)** SEM image of α-Sb_2_S_3_ nanoparticles; **(B)** cycle performance of α-Sb_2_S_3_ at 0.05 A g^−1^; **(C)** SEM image of the few-layer 2D-Sb_2_S_3_ nanosheets; **(D)** cyclic capacity of 2D-SS measured at 0.2 A g^−1^; **(E)** SEM image of Sb_2_S_3_ hollow microspheres; **(F)** cycling performances of three experimental Sb_2_S_3_ electrodes at 1A g^−1^; (g) SEM and TEM images of multi-shell Sb_2_S_3_; **(H)** comparison of the rate performance of multi-shell Sb_2_S_3_, single-shell Sb_2_S_3_, and pristine Sb_2_S_3_. **(A,B)** Adapted with permission from Hwang et al. (2016). Copyright 2013, The Royal Society of Chemistry. **(C,D)** Adapted with permission from Yao et al. (2019). Copyright 2018, Elsevier. **(E,F)** Adapted with permission from Xie et al. (2018). Copyright 2018, Springer. **(G,H)** Adapted with permission from Xie et al. (2019a). Copyright 2019, Elsevier.

Supplementary Figure S2
**(A)** SEM image of Sb_2_S_3_@C rods; **(B)** cycle performance of Sb_2_S_3_@C rods at 0.1 A g^−1^; **(C)** SEM image of SS/CFC; **(D)** cycle performances of SS/CFC and SS powder at 0.5 A g^−1^; **(E)** SEM image of SS/Sb@C nanocomposites; **(F)** cycling performances of SS/Sb@C and Sb_2_S_3_ nanocomposites at 0.1 A g^−1^. **(A,B)** Adapted with permission from Hongshuai Hou et al. (2015). Copyright 2015, American Chemical Society. **(C,D)** Adapted with permission from Liu et al. (2017). Copyright 2017, The Royal Society of Chemistry. **(E,F)** Adapted with permission from Zhao et al. (2020). Copyright 2020, The Royal Society of Chemistry.

Supplementary Figure S3 SEM images: **(A)** In_2_S_3_-Sb_2_S_3_@MCNTs microsphere, **(B)** Sb_2_S_3_/MoS_2_@C composite (SMS@C), **(C)** ZnS-Sb_2_S_3_@C polyhedron, and **(D)** Sb_2_S_3_@FeS_2_/N-graphene composite (SFS/C); **(E)** sodium storage properties of the SMS@C and SMS heterostructure at 5 A g^−1^; **(F)** rate capability of ZnS-Sb_2_S_3_@C core-shell SIB anode; **(G)** schematic illustration of the fabrication process of the SFS/C composite; (h) charge capability of the SFS/C anode at various rates; **(I)** cycle performances of Sb_2_S_3_, SFS, and SFS/C composites at a high rate of 5 A g^−1^. **(A)** Adapted with permission from Huang et al. (2018). Copyright 2018, Wiley-VCH. **(B, E)** Adapted with permission from Wang et al. (2021a). Copyright 2021, Elsevier. **(c,f)** Adapted with permission from Dong et al. (2017). Copyright 2017, American Chemical Society. **(D,G–I)** Adapted with permission from Cao et al. (2020). Copyright 2020, American Chemical Society.

Supplementary Figure S4
**(A)** Schematic diagram of the formation process of the Sb_2_S_3_/MMCN@PPy composite; **(B)** SEM image of Sb_2_S_3_/MMCN@PPy composite; **(C)** rate capability performances of pure Sb_2_S_3_ and Sb_2_S_3_/MMCN@PPy composite; **(D)** schematic illustration of the synthetic process of L-Sb_2_S_3_/Ti_3_C_2_ composite; **(E)** SEM image of L-Sb_2_S_3_/Ti_3_C_2_ composite; **(F)** Rate capability performances of Sb_2_S_3_, Sb_2_S_3_/Ti_3_C_2_, and L-Sb_2_S_3_/Ti_3_C_2_. **(A–C)** Adapted with permission from Yin et al. (2019). Copyright 2019, Elsevier. **(D–F)** Adapted with permission from He et al. (2021). Copyright 2021, Science China Press and Springer-Verlag GmbH Germany, part of Springer Nature.
