# Supplementary material for: Recent Advances in Antimony Sulfide-Based Nanomaterials for High-Performance Sodium-Ion Batteries: A Mini Review
Source: Front Chem. 2022 Apr 7;10:870564. doi: 10.3389/fchem.2022.870564 (PMC9021918; doi:10.3389/fchem.2022.870564)
Supplement: Supplementary file 1 [file DataSheet1.docx]

**Supporting Information**

**Recent Advances in** **Antimony Sulfide-Based Nanomaterials for High-Performance Sodium-Ion Batteries: A Review**

*Guangxin Wang ^a, 1^, Mingyi Guo* *^a, 1,^ *, Yunchao Zhao ^a, b^, Yibo Zhao ^a^, Kun Tang ^a^*, *Zhijun Chen ^c^, Heinz-Rolf Stock ^a^, Yong Liu ^a, b,^ **

^a^ Research Center for High Purity Materials, Henan University of Science and Technology, School of Materials Science and Engineering, Henan University of Science and Technology, Luoyang 471023, P. R. China

^b^ National Joint Engineering Research Center for Abrasion Control and Molding of Metal Materials, Henan Key Laboratory of High-Temperature Structural and Functional Materials, Henan University of Science and Technology, Luoyang 471003, P. R. China

^c^ Luoyang Bearing Research Institute Co. , Ltd. Luoyang 471039, P. R. China

* Corresponding authors and E-mail address:

Mingyi Guo, gmy19910513@163.com;

Prof. Yong Liu, [liuyong209@haust.edu.cn](mailto:liuyong209@haust.edu.cn);

^1^ Mingyi Guo and Guangxin Wang contributed equally to this work.


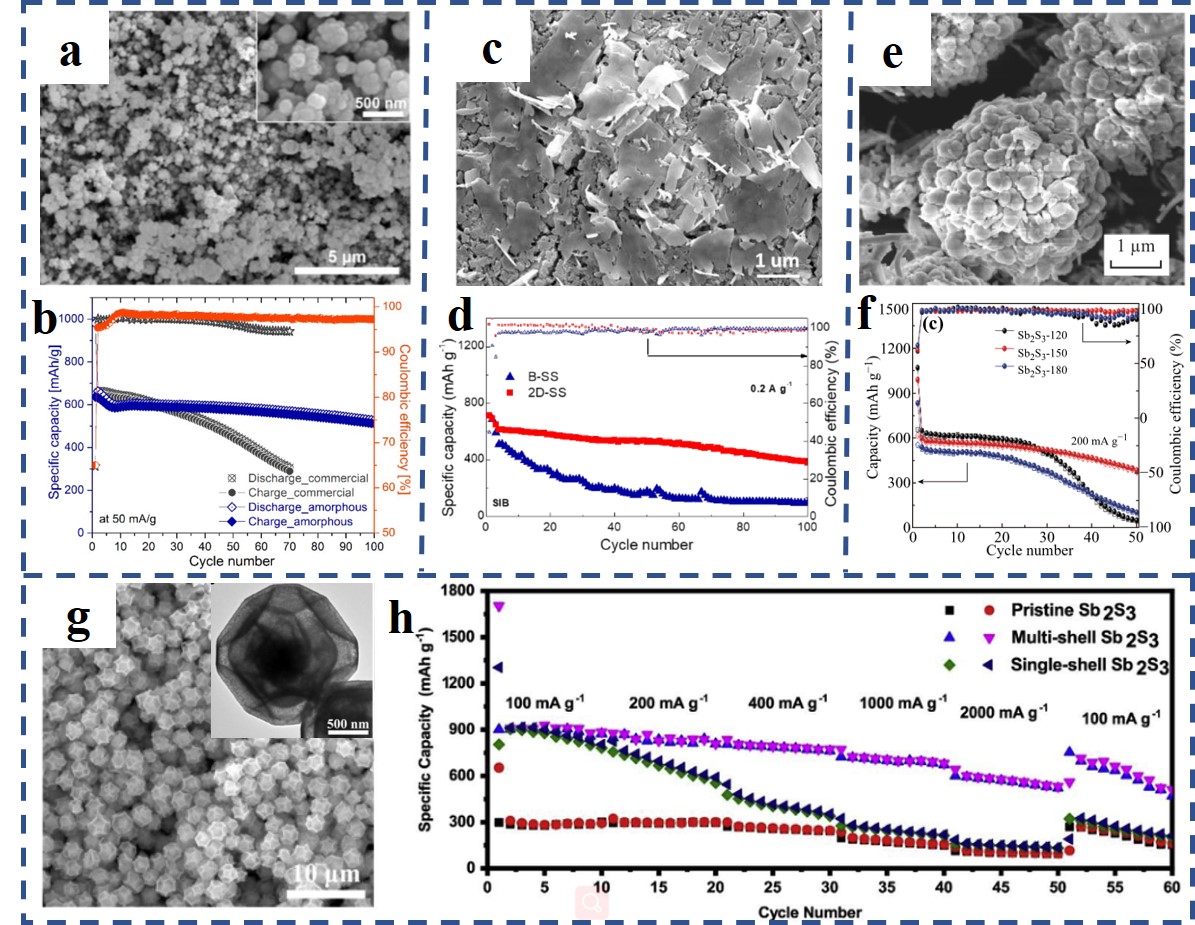


**Fig S1.** (a) SEM image of α-Sb_2_S_3_ nanoparticles; (b) Cycle performance of α-Sb_2_S_3_ at 0.05 A g^-1^; (c) SEM image of the few-layer 2D-Sb_2_S_3_ nanosheets; (d) Cyclic capacity of 2D-SS measured at 0.2 A g^-1^;(e) SEM image of Sb_2_S_3_ hollow microspheres; (f) Cycling performances of three experimental Sb_2_S_3_ electrodes at 1A g^-1^; (g) SEM and TEM images of multi-shell Sb_2_S_3_; (h) Comparison of the rate performance of multi-shell Sb_2_S_3_, single-shell Sb_2_S_3_ and pristine Sb_2_S_3_. **(a,b)** are adapted with permission from Ref. (Hwang et al., 2016). Copyright 2013, The Royal Society of Chemistry. **(c,d)** are adapted with permission from Ref. (Yao et al., 2019). Copyright 2018, Elsevier. **(e,f)** are adapted with permission from Ref. (Xie et al., 2018). Copyright 2018, Springer. **(g,h)** are adapted with permission from Ref. (Xie et al., 2019). Copyright 2019, Elsevier.


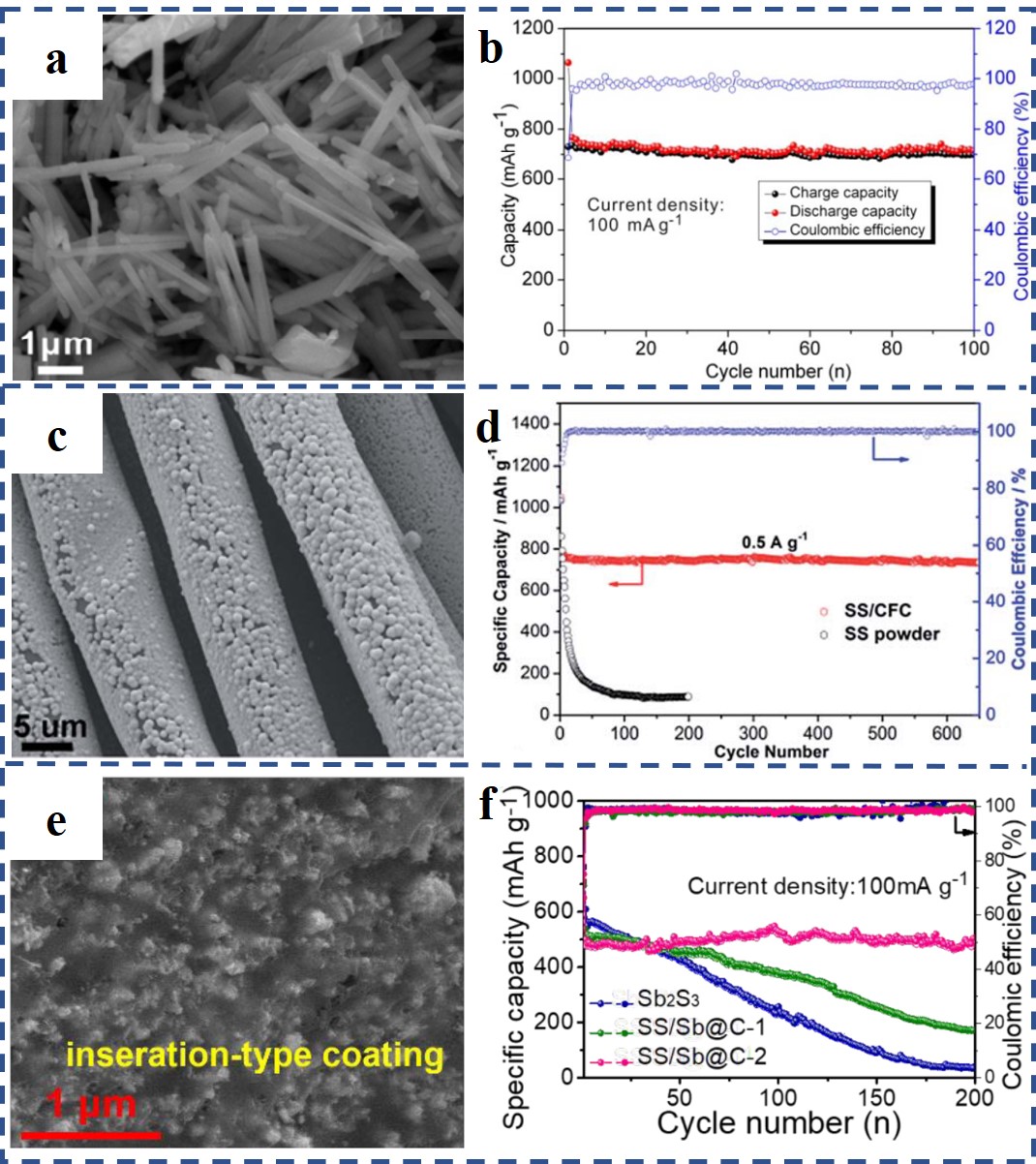


**Fig S2**. (a) SEM image of Sb_2_S_3_@C rods; (b) Cycle performance of Sb_2_S_3_@C rods at 0.1 A g^-1^; (c) SEM image of SS/CFC; (d) Cycle performances of SS/CFC and SS powder at 0.5 A g^-1^; (e) SEM image of SS/Sb@C nanocomposites; (f) Cycling performances of SS/Sb@C and Sb_2_S_3_ nanocomposites at 0.1 A g^-1^. **(a,b)** are adapted with permission from Ref. (Hongshuai Hou, 2015). Copyright 2015, American Chemical Society. **(c,d)** are adapted with permission from Ref. (Liu et al., 2017). Copyright 2017, The Royal Society of Chemistry. **(e,f)** are adapted with permission from Ref. (Zhao et al., 2020). Copyright 2020, The Royal Society of Chemistry.


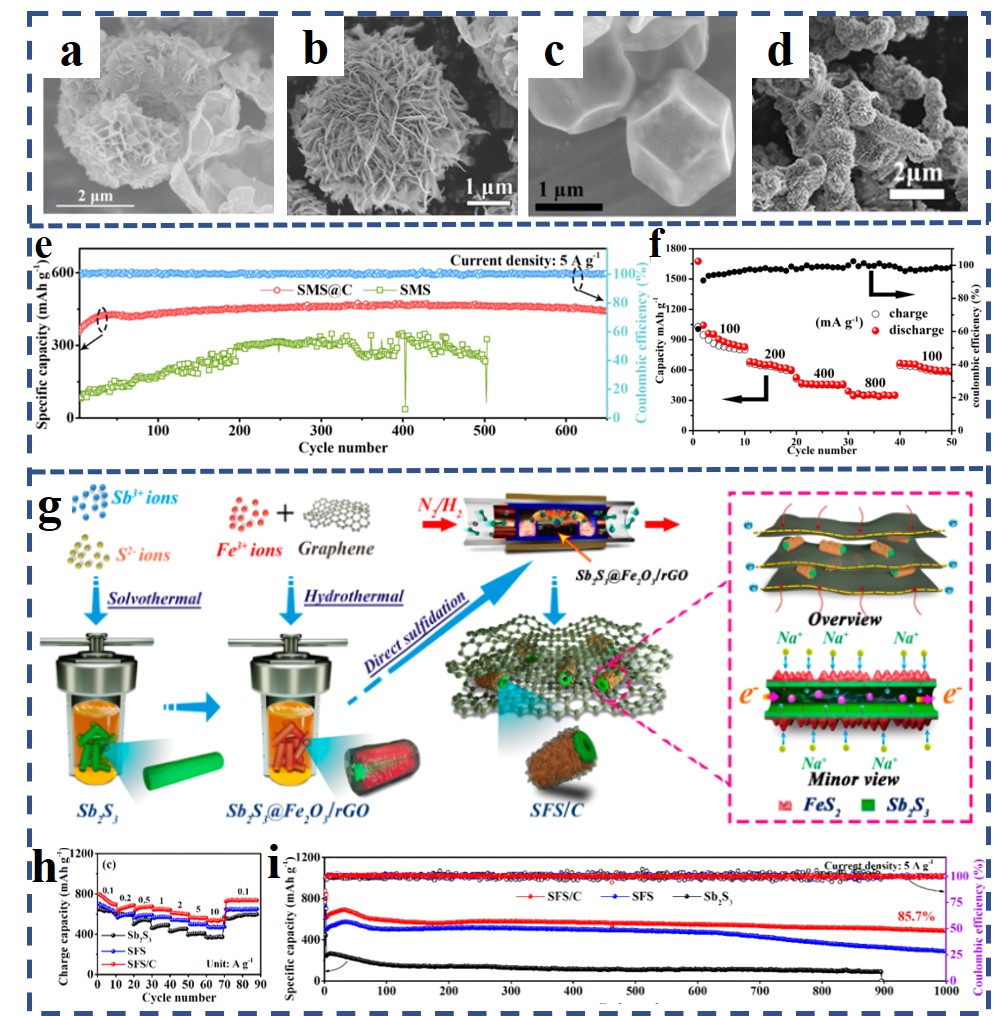


**Fig S3.** SEM images: (a) In_2_S_3_-Sb_2_S_3_@MCNTs microsphere, (b) Sb_2_S_3_/MoS_2_@C composite (SMS@C), (c) ZnS-Sb_2_S_3_@C polyhedron and (d) Sb_2_S_3_@FeS_2_/N-graphene composite (SFS/C ); (e) Sodium storage properties of the SMS@C and SMS heterostructure at 5 A g^-1^; (f) Rate capability of ZnS-Sb_2_S_3_@C core-shell SIBs anode; (g) Schematic illustration of the fabrication process of the SFS/C composite; (h) Charge capability of the SFS/C anode at various rates; (i) Cycle performances of Sb_2_S_3_, SFS, and SFS/C composites at a high rate of 5 A g^-1^. **(a)** is adapted with permission from Ref. (Huang et al., 2018). Copyright 2018, Wiley-VCH . **(b, e)** are adapted with permission from Ref. (Wang et al., 2021). Copyright 2021, Elsevier. **(c, f)** are adapted with permission from Ref. (Shihua Dong, 2017). Copyright 2017, American Chemical Society. **(d, g-i)** are adapted with permission from Ref. (Cao et al., 2020). Copyright 2020, American Chemical Society.


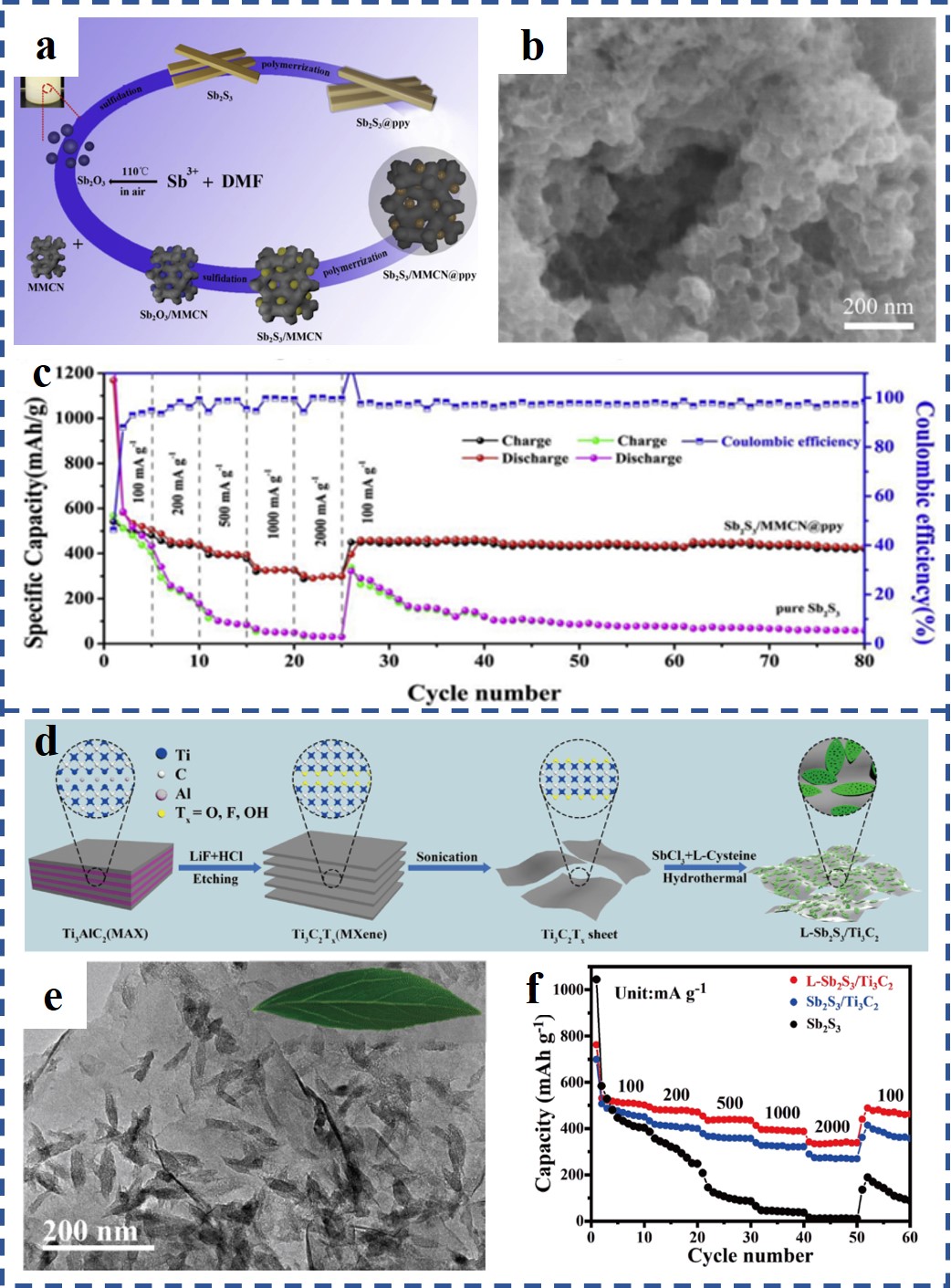


**Fig S4. (a)** Schematic diagram of the formation process of the Sb_2_S_3_/MMCN@PPy composite; **(b)** SEM image of Sb_2_S_3_/MMCN@PPy composite; **(c)** Rate capability performances of pure Sb_2_S_3_ and Sb_2_S_3_/MMCN@PPy composite; **(d)** Schematic illustration of the synthetic process of L-Sb_2_S_3_/Ti_3_C_2_ composite; **(e)** SEM image of L-Sb_2_S_3_/Ti_3_C_2_ composite; **(f)** Rate capability performances of Sb_2_S_3_, Sb_2_S_3_/Ti_3_C_2_, and L-Sb_2_S_3_/Ti_3_C_2_. **(a-c)** are adapted with permission from Ref. (Yin et al., 2019). Copyright 2019, Elsevier. **(d-f)** are adapted with permission from Ref. (He et al., 2021). Copyright 2021, Science China Press and Springer-Verlag GmbH Germany, part of Springer Nature.

**References**

Cao, L., Gao, X., Zhang, B., Ou, X., Zhang, J., and Luo, W.B. (2020). Bimetallic Sulfide Sb2S3@FeS2 Hollow Nanorods as High-Performance Anode Materials for Sodium-Ion Batteries. *ACS Nano* 14(3)**,** 3610-3620. doi: 10.1021/acsnano.0c00020.

He, F., Tang, C., Zhu, G., Liu, Y., Du, A., Zhang, Q., et al. (2021). Leaf-inspired design of mesoporous Sb2S3/N-doped Ti3C2Tx composite towards fast sodium storage. *Science China Chemistry* 64(6)**,** 964-973. doi: 10.1007/s11426-020-9942-9.

Hongshuai Hou, M.J., Zhaodong Huang, Yingchang Yang, Yan Zhang, Jun Chen, Zhibin Wu, and Xiaobo Ji (2015). One-Dimensional Rod-Like Sb2S3-Based Anode for High-Performance Sodium-Ion Batteries. *ACS APPLIED MATERIALS & INTERFACES* 7(34)**,** 19362-19369.

Huang, Y., Wang, Z., Jiang, Y., Li, S., Wang, M., Ye, Y., et al. (2018). Conductivity and Pseudocapacitance Optimization of Bimetallic Antimony-Indium Sulfide Anodes for Sodium-Ion Batteries with Favorable Kinetics. *Adv Sci (Weinh)* 5(10)**,** 1-12. doi: 10.1002/advs.201800613.

Hwang, S.M., Kim, J., Kim, Y., and Kim, Y. (2016). Na-ion storage performance of amorphous Sb2S3nanoparticles: anode for Na-ion batteries and seawater flow batteries. *J. Mater. Chem. A* 4(46)**,** 17946-17951. doi: 10.1039/c6ta07838a.

Liu, S., Cai, Z., Zhou, J., Zhu, M., Pan, A., and Liang, S. (2017). High-performance sodium-ion batteries and flexible sodium-ion capacitors based on Sb2X3(X = O, S)/carbon fiber cloth. *Journal of Materials Chemistry A* 5(19)**,** 9169-9176. doi: 10.1039/c7ta01895a.

Shihua Dong, C.L., Xiaoli Ge, Zhaoqiang Li, Xianguang Miao, and Longwei Yin (2017). ZnS-Sb2S3@C Core-Double Shell Polyhedron Structure Derived from Metal-Organic Framework as Anodes for High Performance Sodium Ion Batteries. *Acs Nano* 11(6)**,** 6474-6482.

Wang, D., Cao, L., Luo, D., Gao, R., Li, H., Wang, D., et al. (2021). Chain mail heterostructured hydrangea-like binary metal sulfides for high efficiency sodium ion battery. *Nano Energy* 87**,** 1-10. doi: 10.1016/j.nanoen.2021.106185.

Xie, F., Zhang, L., Gu, Q., Chao, D., Jaroniec, M., and Qiao, S.-Z. (2019). Multi-shell hollow structured Sb2S3 for sodium-ion batteries with enhanced energy density. *Nano Energy* 60**,** 591-599. doi: 10.1016/j.nanoen.2019.04.008.

Xie, J., Liu, L., Xia, J., Zhang, Y., Li, M., Ouyang, Y., et al. (2018). Template-Free Synthesis of Sb2S3 Hollow Microspheres as Anode Materials for Lithium-Ion and Sodium-Ion Batteries. *Nanomicro Lett* 10(1)**,** 1-12. doi: 10.1007/s40820-017-0165-1.

Yao, S., Cui, J., Deng, Y., Chong, W.G., Wu, J., Ihsan-Ul-Haq, M., et al. (2019). Ultrathin Sb2S3 nanosheet anodes for exceptional pseudocapacitive contribution to multi-battery charge storage. *Energy Storage Materials* 20**,** 36-45. doi: 10.1016/j.ensm.2018.11.005.

Yin, W., Chai, W., Wang, K., Ye, W., Rui, Y., and Tang, B. (2019). A highly Meso@Microporous carbon-supported Antimony sulfide nanoparticles coated by conductive polymer for high-performance lithium and sodium ion batteries. *Electrochimica Acta* 321**,** 1-12. doi: 10.1016/j.electacta.2019.134699.

Zhao, W., Zhang, L., Jiang, F., Chang, X., Yang, Y., Ge, P., et al. (2020). Engineering metal sulfides with hierarchical interfaces for advanced sodium-ion storage systems. *Journal of Materials Chemistry A* 8(10)**,** 5284-5297. doi: 10.1039/c9ta13899d.
